# Reattachment of Fractured Tooth: A Comprehensive Review

**DOI:** 10.7759/cureus.57715

**Published:** 2024-04-06

**Authors:** Pratik Rathod, Nikhil Mankar, Pradnya Nikhade, Manoj Chandak, Aditya Patel, Anuja Ikhar

**Affiliations:** 1 Conservative Dentistry and Endodontics, Sharad Pawar Dental College & Hospital, Datta Meghe Institute of Higher Education & Research, Wardha, IND

**Keywords:** storage media, fragment, crown fracture, reattachment technique, fracture reattachment, traumatic dental injuries

## Abstract

Dental trauma is one of the most prevalent problems encountered in clinical practice. Traumatic injuries involving fractures of the anterior tooth are one of the most common problems among children and adolescents. There is a physical and social impact on patients’ quality of life due to traumatic dental injuries (TDIs). Children and adolescents frequently present with a crown fracture that necessitates immediate intervention. Clinicians need to be aware of various treatment modalities for TDIs and have to address these injuries immediately. Due to advances in adhesive technologies, fragment reattachment is the treatment of choice when the fragment is available and well stored. The purpose of this article is to cover various techniques for reattaching fractured fragments and the most current developments in adhesive systems for this purpose.

## Introduction and background

Approximately 5% of all physical injuries occur in the oral cavity, and traumatic dental injuries (TDIs) account for 92% of patients pursuing treatment for oral injuries [1]. The World Health Organization classifies dental trauma as a public health concern because of its high frequency, which varies from 7.4% to 58% [2,3]. Anterior tooth fractures resulting from trauma are a frequent issue in children and adolescents, with a prevalence of 18.8%. Instances of dental trauma are more common in automobile accidents, contact sports, outdoor activities, and falls [4]. There is a significant risk of dental trauma due to the eruption pattern and position of the upper incisors. TDIs generally impact a patient’s quality of life on a social, psychological, and physical level. Consequently, dental injuries require immediate attention and care to make the fractured tooth functional and aesthetically pleasing.

Crown fracture treatment is based on a number of variables, including the type of fracture, i.e., complicated or uncomplicated, the patient’s age, the degree of severity and nature of the fracture, and the stage of the tooth [5]. For the restoration of fractured teeth, a range of procedures is offered, including composite restorations, post and core, and full coverage crowns [5]. Tooth fragment reattachment, once thought of as an intermediate restoration, has become a recognized treatment modality due to developments in adhesive solutions [6-8]. In 1964, Eidelman and Chosack published the first literature study on reattachment treatment [9]. If a fragment is available and there is an uncomplicated crown fracture in permanent teeth, fragment reattachment is the treatment of choice.

This recommendation is given by the International Association of Dental Traumatology [3]. Some authors have suggested several preparations for the reattachment of broken fragments, while others have suggested “simple reattachment” requiring no additional preparations. Baratieri et al. found fragment reattachment has a number of advantages over traditional acid-etch composite restorations; it is the procedure of choice among clinicians [5].

Since the enamel’s original color, brightness, surface texture, and shape are preserved, improved aesthetics are achieved. A composite restoration will wear down faster than the incisal edge, which also wears at a pace comparable to adjoining teeth. Moreover, this method yields more consistent long-term wear and requires less time. In 1982, Simonsen restored the broken tooth using a “V-shaped, notched bevel” preparation using an acid-etch method and microfilled composite [10]. Simonsen’s technique is the common name for this method. Over time, a number of modifications to the method have been made.

These modifications aim to maintain the reattached fragment’s aesthetics and function, as well as the restoration’s longevity. With recent advances in restorative materials, adhesive protocols, and preparation designs, clinicians can predictably restore fractured teeth. The development of adhesive dentistry has made fragment reattachment simpler and more reliable.

## Review

Advantages of fragment reattachment

The availability of fractured fragments and the incongruity in fit between tooth and fragment are recommendations for fragment reattachment. The procedure is simple, needing minimal time spent chairside; the technique is extremely conservative and involves little to no tooth preparation. In contrast to composite restorations, which abrade more quickly, the incisal edge wears at the same pace as the neighboring tooth. Compared to other comprehensive treatments, it is a cost-effective procedure, and patients who arrive with TDIs experience detrimental psychological effects. Reattachment produces quick results, which instills an overall perception of confidence and optimism; the tooth’s original color, structure, and texture have all been preserved.

Techniques for fragment reattachment

**Figure 1:**
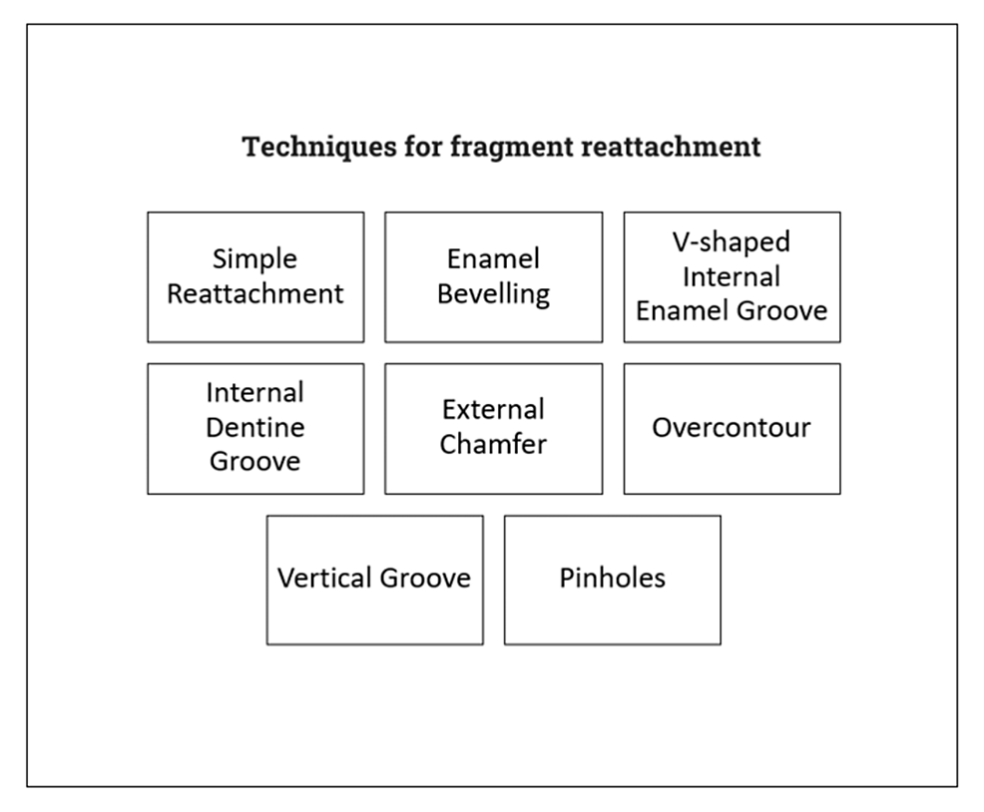
Techniques for fragment reattachment Image credit: Pratik Rathod

Reattaching a fractured tooth has developed into a well-accepted therapeutic technique that consistently produces excellent results [11-13]. There have been numerous proposed changes to tooth reattachment since it was first documented in 1964. Although there is not an exact consensus on which technique produces the best results, some techniques certainly have advantages over others. Figure 1 shows various techniques for fragment reattachment.

Simple Reattachment

Without undergoing any additional fragment preparation, it entails reattaching the fragment. A straightforward reattachment approach can be utilized when the tooth and fragment adapt together without intersegment interference [14]. Studies have revealed that, in comparison to a healthy tooth, reattachment restores 50% of fracture resistance [15]. Srilatha et al. conducted a study on fragment reattachment and found that a simple fragment reattachment procedure recovers fracture strength by nearly 36.6% compared to a sound tooth [16]. Worthington et al., on the other hand, employed a straightforward reattachment method and discovered no variation in the bonding strength between teeth that were reattached without further preparation [17]. It is a comparatively noninvasive procedure and has the benefit of greater aesthetics [18].

Enamel Beveling

Enamel beveling for fragment reattachment was originally put forth by Simonsen in 1979 [19]. This entails a 45° bevel preparation circumferentially on the fragment’s enamel margin as well as on the tooth. In accordance with the author, beveling removes very minimal enamel, and the surface should display the appropriate end-to-end relationship between enamel prisms for the most effective etching and bonding. In 1986, Dean et al. concluded that enamel bevel preparation and simple reattachment had similar retention rates [20]. According to a study conducted by Simonsen in 1979, this technique provides a more appealing appearance because the fracture line has been concealed underneath a layer of composite. Simonsen refuted this claim in 1982 by putting forward the preparation of an internal enamel groove because the composite band on the labial surface had become discolored with time. Another issue is that because the preparation is done before the reattachment, the fragment no longer fits precisely.

V-shaped Internal Enamel Groove

Simonsen, in 1982, first proposed this technique to overcome the drawbacks of the enamel beveling technique [10]. Both the tooth and the fragment have their labial enamel prepared with a V-shaped notch internal bevel, keeping their outer enamel surface intact. Bevel preparation on the palatal surface with a bevel of 45° was advocated by Simonsen. According to a study by Reis et al., creating an internal enamel groove prior to reattachment increases the bond strength by 60% when compared to a straightforward reattachment procedure [21]. The discrepancy in fit is quietly noticeable as preparation is done before reattachment.

Internal Dentine Groove

Before the reattachment procedure, dentinal grooves are made in the fractured fragment as well as in the fractured tooth. The dimensions of the internal dentine groove are 1 mm in depth and 1 mm in width. In a study comparing four distinct reattachment methods, Rajurkar et al. found that the internal dentine groove method demonstrated a bond strength equivalent to that of the enamel beveling method compared to the former [22]. The internal dentinal groove technique of fracture reattachment shows more fracture resistance due to the groove, which may provide more fracture strength and a greater adhesion area. According to Diangelis and Jungbluth, inhibition of eventual darkening because of dentine devitalization in the fragment and an increase in bond strength are provided by an internal dentine groove preparation [23]. In a study comparing four different reattachment techniques, Srilatha et al. found that, in comparison to a sound tooth, the internal dentine groove can regain fracture strength by 89.2% [16]. According to Kulkarni et al., labial double chamfer with lingual overcontouring (54.49%), labial and lingual double chamfer (51.31%), and simple reattachment (28.27%) showed the maximum recovery of fracture resistance. The internal dentinal groove was found to have the highest recovery rate (64.97%) [24].

External Chamfer

Due to prebonding preparation, proper positioning of the fragment may become more challenging if there is a loss of perfect fit between the segments [24]. To overcome this problem, the external chamfer technique was introduced. After the reattachment process, the chamfer is created along the fracture line. In 1983, Davis et al. recommended reattaching the fragment before using a diamond round bur to create an external chamfer in the fracture line, particularly if the area integrating into the fracture line is still visible after a week [25]. In order to assess the fracture strength recovery of anterior fractured tooth fragments that were reattached using various reattachment procedures, Abdulkhayum et al. conducted a study in 2014. They reported fracture strength recoveries of 60.6% and 44.3% for external chamfer and simple reattachment, respectively. These numbers, however, were less than those that came from the overcontour (86.8%) and internal dentinal groove (89.5%) [26].

Overcontour

In the overcontouring technique, there is groove preparation alongside the fracture line, which extends apically and coronally after fragment reattachment. According to Stellini et al., the combined buccal chamfer-lingual contour technique and the overcontour technique can ensure that the restoration’s resistance is at least 50% that of the tooth as a whole [27]. When comparing sound teeth with composite overcontouring, Reis et al. in 2001 showed the highest recovery of fracture strength in their study [21]. Kulkarni et al. in 2022 found the internal dentinal groove had the maximum recovery of fracture resistance (64.97%), followed by the labial double chamfer with lingual overcontour (54.49%), the labial and lingual double chamfer (51.31%), and simple reattachment (28.27%) [24]. A study comparing the bond strength of fractured teeth reattached using four different procedures was carried out by Rajurkar et al. in 2020. They reported that the overcontour fracture reattachment approach showed noticeably higher bond strength when directly compared to the internal dentinal groove, simple reattachment, and enamel beveling technique [22]. Due to a larger adhesion surface, it enables a better composite to be applied to the teeth and encourages a more advantageous allocation of stress in the enamel. However, it has been shown that, with time, resin abrasion causes a progressive loss of aesthetics [18].

Vertical Groove

Using this method, two vertical grooves with a depth and width of two millimeters are created on the tooth’s labial surface following reattachment. The grooves take into account fiber-reinforced composites that are put extra-coronally to aid in fragment retention [28]. Using a vertical groove approach, Karre et al. achieved a 62% fracture strength recovery in proportion to a sound tooth [29].

Pinholes

Using this method, a bilateral pinhole, 1.5 mm in width and depth, is made inside the dentin fragment, 1 mm from the dentin-enamel junction. The pinholes are connected together by a shallow dentinal groove. Beltagy reported that the pinhole technique showed the highest fragment strength as compared to the other techniques used. The in vivo results demonstrated a good aesthetic and functional performance technique. Therefore, pinhole design can be considered an acceptable alternative technique for tooth fragment reattachment in uncomplicated coronal fractures [30].

Factors influencing the longevity of tooth following fracture reattachment

Multiple variables have been observed to influence the long-term viability of fragment reattachment, including reattachment technique, reattachment material, rehydration of the fragment before reattachment, and the existence or lack of an intermediate material. Many authors suggested that the fragment and/or tooth be prepared prior to reattachment [10,19,20,31].

A significant factor in the effectiveness of reattachment is the variety of materials used. Fragments have been reattached using a variety of bonding techniques, including multimodal, self-etch, and total-etch. For fragment reattachment, a variety of intermediary materials have also been employed, including glass ionomer cement, flowable composites, and conventional composites [18,32-34]. According to some authors, the method of reattachment is the primary element influencing the material’s bond strength [21,35]. Conversely, several authors conclude that the bond strength of the reattached fragment depends on both the bonding material and the technique used [2,33,36]. Individually, no method or material can match a sound tooth’s fracture strength, according to Bruschi-Alonso et al. Nonetheless, employing an appropriate reattachment procedure and a suitable bonding technique may enable a reattached tooth to have effective strength when compared to a healthy one [18].

Farik et al. observed that the type of adhesive system used has a direct impact on the fracture strength of the restored tooth [37]. Total-etch adhesives gain significantly more strength after reattachment than self-etch adhesives, according to a study by Bruschi-Alonso et al. investigating the impact strength of these adhesives [18]. Dual-cured, light-cured, or self-cured luting cement, conventional, and flowable composites have all been suggested as intermediate materials. Numerous investigations have shown that the presence of an intermediary material does not directly impact the reattached fragment’s impact strength [18,32]. In 2002, Reis et al. came to the conclusion that the fracture strength of reattached teeth is not significantly influenced by the combination of materials employed to reattach the fragment [32]. Bruschi-Alonso et al. in 2010 used the intermediate material (Rely X CRA or Filtek Z350 Flow) for fractured reattachment and determined that the fracture resistance of the reattached teeth was not affected by the intermediate material [18]. Pusman et al. state that the intermediate material and the bonding adhesive have an impact on the reattached tooth’s fracture strength [2]. Garg et al. found that a flowable composite shows better results than resin cement in fracture reattachment [38].

It has also been demonstrated that the bond strength between the fragment and the tooth is impacted by how the fragment is stored before being reattached. Farik et al. reported that if the reattached fragment is kept dry for more than one hour before the reattachment, the bond strength is diminished. The authors advise preserving the fragment in hydrated media for at least 24 hours before reattaching it if it is kept in a dry environment [39]. Results of a study by Capp et al. show that fracture resistance of the reattached tooth can be restored by preserving a fragment in a hydrated media for 30 minutes before treatment [15]. Prabhakar et al. used media for the storage of fragments, such as coconut water, milk, egg white, or dry air, and found that, among the studied media, milk has the highest values for fracture resistance [40]. According to Hegde and Kale in 2017, fragments kept in milk show better fracture resistance than those observed in a dry environment. Consequently, because milk keeps fragments hydrated, it can be used as an interim storage media [41]. In 2022, Trivedi et al. employed a range of storage media, including dry storage, fresh, tender coconut water, Hanks’ Balanced Salt Solution, milk, and propolis, to store fragments. Their findings indicated that teeth preserved in milk and fresh, tender coconut water exhibited the highest level of fracture resistance. Hence, these two were thought to be preferable storage media [42].

## Conclusions

Clinicians can now use less invasive techniques in their clinical practice because of developments in adhesive technology. Reattaching fractured teeth is a reasonably easy process known as fragment reattachment. Several modifications to the method of preparation have been proposed. However, there is no treatment that can restore the fracture resistance to that of a healthy tooth. However, using the right technique and adhesive material might assist in producing a satisfying result regarding both aesthetics and retention.
